# Appearance of renal hemorrhage in adult mice after inoculation of patient-derived hantavirus

**DOI:** 10.1186/s12985-017-0686-8

**Published:** 2017-01-26

**Authors:** Kenta Shimizu, Takaaki Koma, Kumiko Yoshimatsu, Yoshimi Tsuda, Yuji Isegawa, Jiro Arikawa

**Affiliations:** 1 Department of Microbiology, Hokkaido University Graduate School of Medicine, Kita-15, Nishi-7, Kita-ku, Sapporo 060-8638 Japan; 2 Department of Food Sciences and Nutrition, Mukogawa Women’s University School of Human Environmental Sciences, 6-46 Ikebiraki, Nishinomiya, Hyogo 663-8558 Japan

**Keywords:** Animal model, Hantavirus, Hemorrhagic fever, Mouse, Renal hemorrhage

## Abstract

**Background:**

Hemorrhagic fever with renal syndrome (HFRS) caused by hantavirus infection is characterized by fever, renal dysfunction and hemorrhage. An animal model mimicking symptoms of HFRS remains to be established. In this study, we evaluated the pathogenicity of an HFRS patient-derived Hantaan virus (HTNV) in adult mice.

**Methods:**

Five clones of HTNV strain KHF 83-61 BL (KHFV) that was derived from blood of an HFRS patient were obtained by plaque cloning. The pathogenicity of the virus clones was evaluated by using 6-week-old female BALB/c mice. Sequence analysis of the viral genome was performed by conventional methods.

**Results:**

All of the mice intravenously inoculated with KHFV clone (cl)-1, -2, -3 and -5 showed signs of disease such as transient body weight loss, ruffled fur, reduced activity and remarkably prominent hemorrhage in the renal medulla at 6 to 9 days post-inoculation (dpi) and then recovered. In contrast, mice intravenously inoculated with KHFV cl-4 did not show any signs of disease. We selected KHFV cl-5 and cl-4 as representative of high-pathogenic and low-pathogenic clones, respectively. Quantities of viral RNA in kidneys of KHFV cl-5-infected mice were larger than those in KHFV cl-4-infected mice at any time point examined (3, 6, 9 and 12 dpi). The quantities of viral RNA of KHFV cl-5 and cl-4 peaked at 3 dpi, which was before the onset of disease. Sequence analysis revealed that the amino acid at position 417 in the glycoprotein Gn was the sole difference in viral proteins between KHFV cl-5 and cl-4. The result suggests that amino acid at position 417 in Gn is related to the difference in pathogenicity between KHFV cl-5 and cl-4. When the inoculum of KHFV cl-5 was pretreated with a neutralizing antibody against HTNV strain 76-118, which belongs to the same serotype as KHFV clones, mice did not show any signs of disease, confirming that the disease was caused by KHFV infection.

**Conclusion:**

We found that an HFRS patient-derived HTNV caused renal hemorrhage in adult mice. We anticipate that this infection model will be a valuable tool for understanding the pathogenesis of HFRS.

## Background

Hantaviruses belonging to the family *Bunyaviridae*, genus *Hantavirus* cause two types of disease in humans: hemorrhagic fever with renal syndrome (HFRS) and hantavirus pulmonary syndrome (HPS). HFRS is caused by old world hantaviruses such as Hantaan virus (HTNV), Seoul virus, Dobrava-Belgrade virus and Puumala virus (PUUV) and is characterized by renal dysfunction and hemorrhage. HPS is caused by new world hantaviruses such as Andes virus (ANDV) and Sin Nombre virus (SNV) and is characterized by acute respiratory failure. Although the major symptoms and target organs are different, immune responses and impairment of vascular function are thought to be involved in the pathogenesis of HFRS and HPS [1].

Hantavirus is a spherical enveloped virus with a tri-segmented single-strand negative-sense RNA genome. The segments designated as small (S), medium (M) and large (L) segments encode nucleocapsid protein (N), glycoprotein (GP) and RNA-dependent RNA polymerase (RdRp), respectively. GP is co-translationally cleaved into Gn and Gc.

An animal model is necessary for elucidation of the mechanism of pathogenesis and for development of a vaccine and therapeutic measures. Therefore, many researchers have tried to develop animal models of HPS and HFRS. For HPS, a hamster model using ANDV has been successfully developed [2]. Recently, a non-human primate model using SNV passaged in a deer mouse has also been reported [3]. These models showed acute respiratory distress and severe pulmonary edema, which closely resemble HPS in humans. As for an animal model of HFRS, a non-human primate model using PUUV passaged in voles has been reported, although the animal showed only mild proteinuria, cell filtration and tubular damage in the kidneys, similar to a mild case of HFRS [4]. Various attempts to develop a mouse model of HFRS have also been made. Hantaviruses generally cause asymptomatic infection in adult mice. On the other hand, infection of suckling and newborn mice with hantaviruses causes lethal neurological disease or persistent infection [5, 6], which are different from the disease in humans. We have also tried to develop a mouse model of HFRS. We showed that infection of severe combined immunodeficient mice with HTNV caused lethal wasting disease with pulmonary edema [7, 8]. In addition, we showed that depletion of neutrophils inhibited the development of pulmonary edema. However, there was no hemorrhagic lesion that is characteristic of HFRS [8]. Thus, to date, there has been no animal model showing severe symptoms of HFRS.

As described above, hantaviruses generally cause asymptomatic infection in adult mice. However, lethal adult mice models of HFRS were exceptionally reported by two groups. Wichmann et al. [9] reported that HTNV strain 76-118 caused lethal neurological disease in adult mice. However, the disease was different from HFRS. Asada et al. [10] reported that HTNV strain KHF 83-61 BL (KHFV), which was derived from blood of an HFRS patient, caused lethal infection in adult mice after intravenous inoculation. Although the authors described that the mice showed ruffled fur and hypoactivity, details of the disease remain unclear. In this study, we tried to characterize the pathogenicity of KHFV in mice by using five clones of KHFV and surprisingly found that some clones of KHFV caused renal hemorrhage in adult mice as in HFRS patients.

## Methods

### Viruses and cells

The HTNV strain KHFV was isolated from blood of a patient with Korean hemorrhagic fever by Dr. H. W. Lee and passaged 10 times in the brains of newborn ICR mice [10]. Five clones of KHFV (KHFV cl-1 to -5) were cloned by plaque-purification on Vero E6 cells (ATCC CRL-1586). A stock of KHFV clones was prepared in Vero E6 cells and stored at −80 °C until use. The infectivity titers (focus-forming units; FFU) of the stock of KHFV clones were measured by counting infected cell foci detected by immunostaining as described previously [11].

### Pathogenicity in mice

Female BALB/cAJcl mice (6 weeks old) were purchased from CLEA Japan Inc. Mice inoculated with 10^5^ FFU of KHFV cl-1 to -5 via the intraperitoneal (i.p.) or intravenous (i.v.) route were observed for 2 weeks and then euthanized and subjected to autopsy. In another experiment, kidneys of mice intravenously inoculated with 10^5^ FFU of KHFV cl-4 and cl-5 were collected at 3, 6, 9 and 12 days post-inoculation (dpi) and subjected to quantification of viral RNA and pathological examination. In experiments for examination of dose dependency, serial 10-fold dilutions of KHFV cl-5 (10^2^ to 10^5^ FFU) were intravenously inoculated into mice, and the mice were observed for 2 weeks. In experiments for examination of the specificity of virus in disease development, 10^5^ FFU of KHFV cl-5 were pretreated with 8-fold dilutions of sera of HTNV strain 76-118-infected mice (neutralizing antibody titer: 640) [6] or normal mice at 37 °C for 1 h and intravenously inoculated into mice.

### Quantification of viral RNA

Total RNAs extracted from kidney homogenates of infected mice were reverse-transcribed into cDNA by using random primers and SuperScript II Reverse Transcriptase (Thermo) according to the manufacturer’s instructions. The cDNAs were subjected to real-time PCR analysis using primers KHFV-SF (5′-TGGACCAAAGGATTATTGTGC-3′) and HTNV-SR (5′-CATCCCCTAAGTGGAAGTTGTC-3′), Universal Probe Library #75, LightCycler 480 Probes Master and LightCycler 480 Instrument II (Roche) according to the manufacturer’s instructions. The open reading frame (ORF) of the NP gene of KHFV cl-5 was amplified by PCR using primers HTN-NP1F-EcoRI (5′-AGAGAATTCATGGCAACTATGGAGGAATTAC-3′) and HTN-NP429R-XhoI (5′-ATTCTCGAGTTAGAGTTTCAAAGGCTCTTG-3′) and Platinum Taq DNA Polymerase High Fidelity (Thermo) and then cloned into the pGEM T vector (Promega) and used as a standard in real-time PCR.

### Nucleotide sequencing

Total RNAs extracted from Vero E6 cells infected with KHFVs were reverse-transcribed into cDNA as described above. The cDNAs covering the entire viral genome were amplified by PCR using primers and Platinum Taq DNA Polymerase High Fidelity (Thermo) according to the manufacturer’s instructions. We amplified cDNAs including 3′ and 5′ terminal non-coding regions of each segment by adding adaptor to the 3′ end of genomic and anti-genomic RNAs as described in a previous study [12]. Information on the primers is available on request. The PCR products were purified with a MinElute PCR Purification Kit or a MinElute Gel Extraction Kit (Qiagen) and subjected to direct sequencing using a BigDye Terminator v3.1 Cycle Sequencing Kit and Applied Biosystems 3130xl Genetic Analyzer (Thermo) according to the manufacturer’s instructions. Sequences were analyzed by Genetyx-Mac version 15.0.1 (Genetyx Corp.).

### Statistical analysis

Student’s *t* test was used for comparison of quantities of viral RNA. A *P* value <0.05 was considered statistically significant.

## Results

### Pathogenicity of KHFV clones in mice

**Fig. 1:**
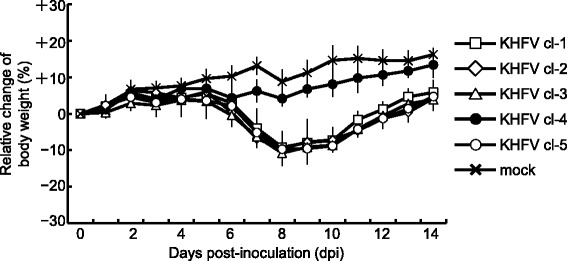
Changes in body weight of KHFV-infected mice. Mice were intravenously inoculated with 10^5^ FFU of KHFV cl-1 to -5 and observed for 2 weeks. Body weight of mice at the time of inoculation was set as baseline, and percent changes from the baseline are shown. The values are averages and standard deviations of percent changes

In order to examine the pathogenicity of KHFV cl-1 to -5 in mice, 10^5^ FFU each of the viruses were inoculated into mice via the i.p. or i.v. route. The mice inoculated with the viruses via the i.p. route did not develop any signs of disease except for one of the three mice inoculated with KHFV cl-5, which developed signs of disease such as body weight loss, ruffled fur and decrease in movement at 6 to 9 dpi and then recovered (Table 1). In contrast, when the viruses were inoculated via the i.v. route, although the mice inoculated with KHFV cl-4 did not develop any signs of disease, all of the mice inoculated with KHFV cl-1, -2, -3 and -5 developed signs of disease at 6 to 9 dpi (Table 1). Relative changes in body weight of mice inoculated with KHFV cl-1 to -5 via the i.v. route are shown in Fig. 1. Body weight of the mice inoculated with KHFV cl-4 gradually increased as did that of mock-infected mice. In contrast, body weight of the mice inoculated with KHFV cl-1, -2, -3 and -5 decreased by about 10% at around 6 to 9 dpi and then increased. We selected KHFV cl-5 and cl-4 as representative of high-pathogenic and low-pathogenic clones, respectively.

**Table 1:** Morbidity in mice infected with KHFV cl-1 to -5

Virus^a^	Route of infection	
	i.p.	i.v.
KHFV cl-1	0/2^b^	3/3
KHFV cl-2	0/2	3/3
KHFV cl-3	0/3	3/3
KHFV cl-4	0/3	0/3
KHFV cl-5	1/3	3/3

^a^10^5^ FFU/mouse

^b^Number of mice showing transient weight loss and ruffled fur/Number of mice inoculated

### Quantity of viral RNA in the kidney

**Fig. 2:**
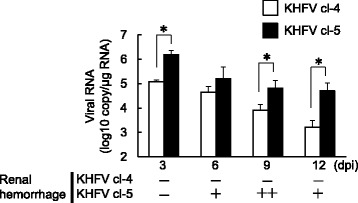
Quantities of viral RNA in kidneys of KHFV-infected mice. Mice were intravenously inoculated with 10^5^ FFU of KHFV cl-4 and cl-5. Quantities of viral RNA in kidneys of mice at 3, 6, 9 and 12 days post-inoculation were determined by real-time PCR. The values are averages and standard deviations of the copy number of viral RNA. Asterisks indicate a significant difference in quantity of viral RNA (*P* < 0.05)

To determine whether viral load in the kidney was correlated with disease progression, the quantity of viral RNA in kidneys of mice inoculated with 10^5^ FFU of KHFV cl-4 or cl-5 via the i.v. route was examined by real-time PCR. The quantity of viral RNA of KHFV cl-5 was greatest at 3 dpi, which was before the onset of disease, and gradually decreased (Fig. 2). Similar results were obtained from analysis of KHFV cl-4-infected mice. However, the copy numbers of viral RNA of KHFV cl-5 were larger than those of KHFV cl-4 at any time point. In the process of sample collection, we found that kidneys of mice inoculated with KHFV cl-5, but not mice inoculated with KHFV cl-4, contain hemorrhagic lesions in the renal medulla. Then we conducted pathological examination of the kidneys of mice inoculated with KHFV cl-5.

### Pathological changes in kidneys

**Fig. 3:**
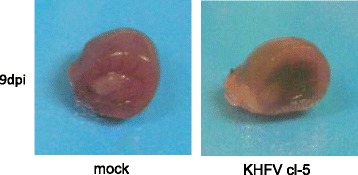
Images of kidneys of KHFV cl-5-infected mice at 9 days post-inoculation. Kidneys of mice intravenously inoculated with 10^5^ FFU of KHFV cl-5 were subjected to pathological examination. There were three mice per group. Representative images are shown

**Fig. 4:**
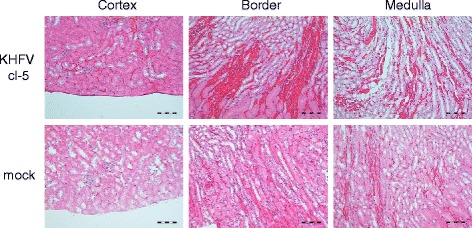
Histopathological images of the kidneys of KHFV cl-5-infected mice at 9 days post-inoculation. Kidneys of mice intravenously inoculated with 10^5^ FFU of KHFV cl-5 were subjected to histopathological examination. There were three mice per group. Representative images are shown

Mice were inoculated with 10^5^ FFU of KHFV cl-5 via the i.v. route, and kidneys were collected from three mice each at 3, 6, 9 and 12 dpi and subjected to pathological examination. There was no pathological change at 3 dpi. However, after 6 dpi, macroscopic hemorrhagic lesions began to be observed in the renal medulla of KHFV cl-5-infected mice (Figs. 2 and 3). The severity of hemorrhage peaked at 9 dpi and was weakened at 12 dpi. Histopathological examination showed that congestion and hemorrhage were prominent in the border between the renal cortex and medulla (Fig. 4). A flattened tubular epithelium, dilated lumina and infiltrating cells were observed in the renal medulla (Fig. 4). These pathological changes after 6 dpi were observed in all of the infected mice.

### Dose-dependency of the pathogenicity in mice

To determine whether KHFV cl-5 causes disease in mice in a dose-dependent manner, serial 10-fold dilutions of the virus (10^5^ to 10^2^ FFU) were inoculated into each of 7 mice via the i.v. route and the mice were observed for 2 weeks. All of the mice inoculated with 10^5^ FFU and 10^4^ FFU of KHFV cl-5 developed signs of disease, while none of the mice inoculated with 10^3^ and 10^2^ FFU of the virus showed signs of disease (Table 2). The dose causing disease in 50% of mice was calculated to be 3.2 × 10^3^ FFU by the method of Reed and Muench [13].

**Table 2:** Morbidity in mice intravenously inoculated with different doses of KHFV cl-5

Dose (FFU)	Morbidity
10^5^	7/7^a^
10^4^	7/7
10^3^	0/7
10^2^	0/7

^a^Number of mice showing transient weight loss and ruffled fur/number of mice inoculated

### Specificity of virus in disease development

**Fig. 5:**
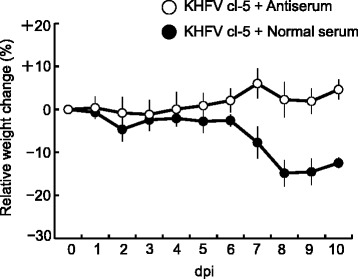
Changes in body weight of mice inoculated with neutralizing antibody-treated KHFV cl-5. The inoculum of KHFV cl-5 (10^5^ FFU) was pretreated with a neutralizing antibody against HTNV strain 76-118 or with normal mouse serum at 37 °C for 1 h. Body weight of mice at the time of inoculation was set as baseline, and percent changes from the baseline are shown. The values are averages and standard deviations of percent changes

To confirm that the disease was caused by KHFV infection and not by unknown etiology, 10^5^ FFU of KHFV cl-5 was pretreated with a neutralizing antibody to HTNV strain 76-118, which is the same serotype as KHFVs, or with normal mouse serum for 1 h at 37 °C and then inoculated into mice via the i.v. route. The mice inoculated with the virus pretreated with normal mouse serum showed transient body weight loss around 6 to 9 dpi. In contrast, the mice inoculated with the virus pretreated with the neutralizing antibody developed no signs of disease (Fig. 5). Pathological examination showed that renal hemorrhage occurred at 10 dpi in mice inoculated with the virus pretreated with normal mouse serum but did not occur in mice inoculated with the virus pretreated with the neutralizing antibody. These results indicate that the disease development was caused by KHFV infection.

### Comparison of viral genome sequences of KHFV cl-4 and cl-5

To reveal the differences in genetic background between KHFV cl-4 and cl-5, which showed different virulency, genome sequences were determined and compared. In the ORF of the N gene, there was no difference between KHFV cl-4 and cl-5. In the ORF of the GP gene, the nucleotide at position 1249 was different, causing an amino acid difference at position 417 in the glycoprotein Gn: lysine for KHFV cl-4 and glutamic acid for KHFV cl-5 (Table 3). In the ORF of RdRp gene, the nucleotide at position 6363 was different, but it did not cause an amino acid difference. The results indicate that the amino acid at position 417 in the glycoprotein Gn is the sole amino acid difference in viral proteins between KHFV cl-4 and cl-5. The amino acids at position 417 in Gn of KHFV cl-1, −2 and −3 were the same as that in Gn of KHFV cl-5. In the non-coding regions of S, M and L segments, there was no difference between KHFV cl-4 and cl-5.

**Table 3:** Differences in sequences of viral genes between KHFV cl-4 and −5

Virus	Nucleocapsid protein		Glycoprotein		Polymerase	
	nt^a^	aa^b^	nt 1249	aa 417	nt 6363	aa
KHFV cl-4	–^c^	–	A	Lys	G	–
KHFV cl-5	–	–	G	Glu	A	–

^a^Nucleotide sequence

^b^Deduced amino acid sequence

^c^No Difference

## Discussion

In this study, we found KHFV clones causing renal hemorrhage in adult BALB/c mice after inoculation via the i.v. route. The development of disease was inhibited by pretreatment of an inoculum with a neutralizing antibody against HTNV strain 76-118, which is the same serotype as KHFV clones, indicating that the disease was caused by KHFV infection. Renal hemorrhagic lesions in the mice were macroscopically limited to the medullary region, which is one of the characteristic pathological changes in severe HFRS patients [14]. Widespread capillary engorgement, interstitial hemorrhage, flattened tubular epithelium, dilated lumina and infiltrating cells in the renal medullary region in the mice have also been observed in HFRS patients [15, 16]. These results indicate that the pathological lesion that developed in the kidney of KHFV-infected mice was similar to that in HFRS patients.

Immune responses are thought to be related to the pathogenesis of hantavirus infection. In HFRS patients, increased numbers of CD8+ T cells and a decrease in the ratio of CD4+ T cells to CD8+ T cells have been reported [17]. In HPS patients, high levels of T cells and cytokine-producing cells were found in lungs [18], and T cell numbers have been suggested to correlate with disease severity [19]. In our mouse model, cells infiltrating the renal medulla were observed in the KHFV-infected mice. Importance of viral propagation in disease severity has also been reported. Yi et al. [20] reported that HFRS patients in a severe group had higher viral loads in the early stage of disease than did those in a mild/moderate group. We also showed that the quantity of viral RNA in kidneys of KHFV cl-5-infected mice was larger than those of KHFV cl-4-infected mice and peaked before the onset of disease. The initial virus propagation may excessively induce host immunity and cause disease afterward. Alternatively, there is a possibility that damage caused by the initial virus propagation was sufficient to ultimately result in disease development despite onset of virus clearance by the immune response. It would be interesting to examine the number of T cells in the kidney and the role of T cells in the pathogenesis in KHFV-infected mice in further studies.

Although Asada et al. [10] reported that KHFV killed adult mice after intravenous inoculation, we could not reproduce the lethal outcome by using KHFV clones for unknown reasons. However, KHFV clones caused renal hemorrhage in adult mice. Because of the unavailability of the same virus and mice as those used by Asada et al., we could not examine the reason for the different outcomes. Instability of character of hantavirus has been reported in previous studies. Wichmann et al. [9] reported that HTNV strain 76-118 caused lethal neurological disease in adult laboratory mice. However, the result could not be reproduced in other laboratories. Klingstrom et al. [21] reported that mice inoculated with cell culture-passaged PUUV showed lower IgG production compared to wild-type PUUV. Safronetz et al. [3] reported that deer mouse-only-passaged SNV, but not Vero cell-passaged SNV, caused severe respiratory disease in rhesus macaques. Therefore, there is a possibility that characters of KHFV clones are changed by passages in some tissues and cells. Although we confirmed that the phenotype of KHFV cl-5 was maintained after three times of passages in Vero E6 cells, it will be important to prepare virus stock with lower number of passages and to confirm the character of the virus stock after preparation.

We found that KHFV cl-4 did not cause disease in mice and that the amino acid at position 417 in the glycoprotein Gn is the sole amino acid difference in viral proteins between KHFV cl-4 and cl-5. The amino acid at position 417 in Gn of KHFV cl-1 to -3 was the same as that of KHFV cl-5. These results suggest that the amino acid at position 417 in Gn is involved in the difference in pathogenicity between KHFV cl-4 and cl-5. However, analysis of database showed that almost all of the HTNV strains have an amino acid shared by virulent clones, although HTNV strains generally cause no disease in adult mice. Therefore, other factors should be involved in the difference of pathogenicity between KHFV cl-5 and other HTNV strains. Because the amino acid at position 417 was located in the ectodomain of Gn, the amino acid difference may affect the function of Gn such as binding to and entry into host cells. In fact, the quantity of viral RNAs in kidneys of KHFV cl-4-infected mice was smaller than that in KHFV cl-5-infected mice at any time point. The difference in viral propagation between KHFV cl-4 and cl-5 may lead to the difference in pathogenicity. Further studies are needed to elucidate the mechanism by which the amino acid at position 417 is involved in the pathogenesis.

## Conclusions

We found KHFV clones causing renal hemorrhage in adult mice, which is similar to that in HFRS patients. Closely related viral clones with different pathogenicities were obtained. In addition, a single amino acid difference in viral proteins between high-pathogenic and low-pathogenic clones was identified. Although it is necessary to examine whether the mouse model shares some mechanisms of the pathogenesis in humans, we anticipate that this infection model will improve the understanding of HFRS pathogenesis.

## Abbreviations

aa: Deduced amino acid sequence
ANDV: Andes virus
cl: Clone
dpi: Days post-inoculation
FFU: Focus-forming units
GP: Glycoprotein
HFRS: Hemorrhagic fever with renal syndrome
HPS: Hantavirus pulmonary syndrome
HTNV: Hantaan virus
i.p.: Intraperitoneal
i.v.: Intravenous
KHFV: KHF 83-61 BL
L: Large
M: Medium
N: Nucleocapsid protein
nt: Nucleotide sequence
ORF: Open reading frame
PUUV: Puumala virus
RdRp: RNA-dependent RNA polymerase
S: Small
SNV: Sin Nombre virus

## References

[1] Jonsson CB, Figueiredo LTM, Vapalahti O (2010). A global perspective on hantavirus ecology, epidemiology, and disease. Clin Microbiol Rev.

[2] Hooper JW, Larsen T, Custer DM, Schmaljohn CS (2001). A lethal disease model for hantavirus pulmonary syndrome. Virology.

[3] Safronetz D, Prescott J, Feldmann F, Haddock E, Rosenke R, Okumura A, Brining D, Dahlstrom E, Porcella SF, Ebihara H, Scott DP, Hjelle B, Feldmann H (2014). Pathophysiology of hantavirus pulmonary syndrome in rhesus macaques. Proc Natl Acad Sci U S A.

[4] Groen J, Gerding M, Koeman JP, Roholl PJM, Amerongen GV, Jordans HGM, Niesters HGM, Osterhaus ADME (1995). A macaquemodel for hantavirus infection. J Infect Dis.

[5] Yoo YC, Yoshimatsu K, Yoshida R, Tamura M, Azuma I, Arikawa J (1993). Comparison of virulence between Seoul virus strain SR-11 and Hantaan virus strain 76–118 of hantaviruses in newborn mice. Microbiol Immunol.

[6] Araki K, Yoshimatsu K, Lee BH, Kariwa H, Takashima I, Arikawa J (2003). Hantavirus-specific CD8 + −T-cell responses in newborn mice persistently infected with Hantaan virus. J Virol.

[7] Yoshimatsu K, Arikawa J, Ohbora S, Itakura C (1997). Hantavirus infection in SCID mice. J Vet Med Sci.

[8] Koma T, Yoshimatsu K, Nagata N, Sato Y, Shimizu K, Yasuda SP, Amada T, Nishio S, Hasegawa H, Arikawa J (2014). Neutrophil depletion suppresses pulmonary vascular hyperpermeability and occurrence of pulmonary edema caused by hantavirus infection in C.B-17 SCID mice. J Virol.

[9] Wichmann D, Grone HJ, Frese M, Pavlovic J, Anheier B, Haller O, Klenk HD, Feldmann H (2002). Hantaan virus infection causes an acute neurological disease that is fatal in adult laboratory mice. J Virol.

[10] Asada H, Balachandra K, Tamura M, Kondo K, Yamanishi K (1989). Cross-reactive immunity among different serotypes of virus causing haemorrhagic fever with renal syndrome. J Gen Virol.

[11] Araki K, Yoshimatsu K, Ogino M, Ebihara H, Lundkvist A, Kariwa H, Takashima I, Arikawa J (2001). Truncated hantavirus nucleocapsid proteins for serotyping Hantaan, Seoul, and Dobrava hantavirus infections. J Clin Microbiol.

[12] Ito N, Kakemizu M, Ito KA, Yamamoto A, Yoshida Y, Sugiyama M, Minamoto N (2001). A comparison of complete genome sequences of the attenuated RC-HL strain of rabies virus used for production of animal vaccine in Japan, and the parental Nishigahara strain. Microbiol Immunol.

[13] Reed LJ, Muench H (1983). A simple method of estimating fifty percent points. Am J Hyg.

[14] Lee M (1983). Korean Hemorrhagic fever.

[15] Collan Y, Mihatsch MJ, Lahdevirta J, Jokinen EJ, Romppanen T, Jantunen E (1991). Nephropathia epidemica: mild variant of hemorrhagic fever with renal syndrome. Kidney Int.

[16] Mustonen J, Helin H, Pietila K, Brummer-Korvenkontio M, Hedman K, Vaheri A, Pasternack A (1994). Renal biopsy findings and clinicopathologic correlations in nephropathia epidemica. Clin Nephrol.

[17] Chen LB, Yang WS (1990). Abnormalities of T cell immunoregulation in hemorrhagic fever with renal syndrome. J Infect Dis.

[18] Mori M, Rothman AL, Kurane I, Montoya JM, Nolte KB, Norman JE, Waite DC, Koster FT, Ennis FA (1999). High levels of cytokine-producing cells in the lung tissues of patients with fatal hantavirus pulmonary syndrome. J Infect Dis.

[19] Kilpatrick ED, Terajima M, Koster FT, Catalina MD, Cruz J, Ennis FA (2004). Role of specific CD8+ T cells in the severity of a fulminant zoonotic viral hemorrhagic fever, hantavirus pulmonary syndrome. J Immunol.

[20] Yi J, Xu Z, Zhuang R, Wang J, Zhang Y, Ma Y, Liu B, Zhang Y, Zhang C, Yan G, Zhang F, Xu Z, Yang A, Jin B (2013). Hantaan virus RNA load in patients having hemorrhagic fever with renal syndrome: correlation with disease severity. J Infect Dis.

[21] Klingstrom J, Hardestam J, Lundkvist A (2006). Dobrava, but not Saaremaa, hantavirus is lethal and induces nitric oxide production in suckling mice. Microbes Infect.

